# Lie Detection Using fNIRS Monitoring of Inhibition-Related Brain Regions Discriminates Infrequent but not Frequent Liars

**DOI:** 10.3389/fnhum.2018.00071

**Published:** 2018-03-13

**Authors:** Fang Li, Huilin Zhu, Jie Xu, Qianqian Gao, Huan Guo, Shijing Wu, Xinge Li, Sailing He

**Affiliations:** ^1^Centre for Optical and Electromagnetic Research, South China Academy of Advanced Optoelectronics, South China Normal University (SCNU), Guangzhou, China; ^2^College of Teacher Education and Psychology, Sichuan Normal University, Chengdu, China; ^3^School of Psychology, South China Normal University (SCNU), Guangzhou, China; ^4^Guangdong Dance and Drama College, Foshan, China; ^5^Department of Electromagnetic Engineering, Royal Institute of Technology, Stockholm, Sweden

**Keywords:** fNIRS, deception, detection feasibility, inhibition, middle frontal gyrus

## Abstract

Functional near-infrared spectroscopy (fNIRS) was used to test whether monitoring inhibition-related brain regions is a feasible method for detecting both infrequent liars and frequent liars. Thirty-two participants were divided into two groups: the deceptive group (liars) and the non-deceptive group (ND group, innocents). All the participants were required to undergo a simulated interrogation by a computer. The participants from the deceptive group were instructed to tell a mix of lies and truths and those of the ND group were instructed always to tell the truth. Based on the number of deceptions, the participants of the deceptive group were further divided into a infrequently deceptive group (IFD group, infrequent liars) and a frequently deceptive group (FD group, frequent liars). The infrequent liars exhibited greater neural activities than the frequent liars and the innocents in the left middle frontal gyrus (MFG) when performing the deception detection tasks. While performing deception detection tasks, infrequent liars showed significantly greater neural activation in the left MFG than the baseline, but frequent liars and innocents did not exhibit this pattern of neural activation in any area of inhibition-related brain regions. The results of individual analysis showed an acceptable accuracy of detecting infrequent liars, but an unacceptable accuracy of detecting frequent liars. These results suggest that using fNIRS monitoring of inhibition-related brain regions is feasible for detecting infrequent liars, for whom deception may be more effortful and therefore more physiologically marked, but not frequent liars.

## Introduction

Functional near-infrared spectroscopy (fNIRS) is an advanced technique which can detect the neural signals of the cortical regions of the brain (Tsuzuki and Dan, 2014). fNIRS has competitive temporal resolution and spatial resolution compared with other techniques (Zhu et al., 2015). Additionally, fNIRS costs less (Naseer and Hong, 2015; Yücel et al., 2015), and can be used in less controlled environments (Pinti et al., 2015). Recently, fNIRS has been increasingly used in assessing the neural activities in social cognition (Naseer and Hong, 2013; Naseer et al., 2014, 2016a,b), such as deception (Hu X. S. et al., 2012; Vega et al., 2016).

Deception is a cognitive process defined as intentionally suppressing the truth and producing false responses to obtain rewards or to avoid punishments (Spence et al., 2001; Ganis et al., 2009). Generally, deception has been consistently recognized as more cognitively demanding than telling the truth (Blandón-Gitlin et al., 2014; Gamer, 2014; Gawrylowicz et al., 2016), because deceiving requires more cognitive resources to process the risk or reward calculation, to execute the plans, to speculate on others’ ideas, to inhibit the truth and to produce the new responses in a clever way (Sip et al., 2008; Spence et al., 2008; Christ et al., 2009; Leue et al., 2012; Ding et al., 2014). Consequently, deception often leads to greater neural responses compared to telling the truth (Sip et al., 2008; Ganis et al., 2009; Gamer, 2014), which could make deception detection feasible. Among various cognitive activities during deception, inhibiting the truth plays a central role (Verschuere et al., 2012; Debey et al., 2015). The function of inhibition is closely linked to the neural activities of the prefrontal cortex, especially related to the activities of the left middle frontal gyrus (MFG) and the bilateral inferior frontal gyrus (IFG; Jonides et al., 1998; Aron et al., 2003; Swick et al., 2008; Marchewka et al., 2012; Sip et al., 2013). Existing studies show empirical evidence that these regions involved in inhibition could be significantly activated during different kinds of deception (Browndyke et al., 2008; Ito et al., 2011; Marchewka et al., 2012; Proverbio et al., 2013). For instance, Marchewka et al. (2012) proved that significantly greater activation of the bilateral IFG could be observed whether lying about general information or about individual information than telling the truth. In addition, Ito et al. (2011) found that deceiving in response to neutral events and to emotional events were both associated with more neural activation of left MFG than telling the truth. These studies all suggest that inhibition-related brain regions are a feasible index for detecting deception.

However, less attention has been paid to how individual differences could affect the neural activation associated with the deception. One significant factor is the frequency of deception. In real life, frequent deception offers individuals more opportunities for training themselves in deceptive skills, which makes their deceiving proficient (Jiang et al., 2013). Thus, deception would become a relatively automatic and dominant response for frequent liars (Hu X. et al., 2012; Jiang et al., 2013), that is, frequent deception makes their deceiving easier. Several studies have demonstrated this phenomenon. For example, two studies indicated that frequent deception made the process of deceiving less wrong (Verschuere et al., 2011; Van Bockstaele et al., 2012). Importantly, as deception becomes easier, it is reasonable to speculate that the neural responses of deception, particularly neural activities of inhibition-related areas, would decrease. For instance, Jiang et al. (2013) showed that the neural activities of the left MFG during strategic deception were reduced in frequent liars compared to infrequent liars. This phenomenon could pose a challenge to the application of lie detection using the inhibition-related regions as an index, as it suggests that it might fail to find greater neural responses during deception compared to telling the truth for frequent liars. Also, it suggests that distinguishing the individuals who are constant liars from innocents might become harder. The effect of frequency of deception on the detection feasibility of the deception is an important issue for real world lie-detector systems.

Using brain areas involved in inhibition (the left MFG and the bilateral IFG) as the regions of interests (ROIs), we aim to ascertain whether inhibition-related regions are a feasible index for detecting both infrequent liars and frequent liars. We not only examined the effect of frequency of deception on the neural activities associated with deception by group analysis, but also investigated the effect of frequency of deception on the accuracy of detecting different liars by individual analysis. Feasible detection requires two results: (1) in the group analysis, liars showed significantly greater neural activities compared to baseline during deceiving (as the previous studies showed, baseline was often set as the task of telling the truth without any other motivation Gamer, 2014), while innocents exhibited distinct neural activity patterns when performing deception detection tasks; and (2) in the individual analysis, acceptable accuracy could be obtained in differentiating the liars from innocents. We defined acceptable accuracy as “successful differentiation between liars and innocents with at least 70% accuracy” (from one review by Gamer, 2014, the average accuracy of deception detection from several typical studies was above 70%).

This study addresses three poorly understood aspects of lie detection: it raises the issue of the limitations of using inhibition-related brain regions to detect deception, which could initially explore whether simple neural indices could be used to detect deception for various populations. It analyses the accuracy of detecting two types of deceptive individuals (frequent liars and infrequent liars). The results of the detection accuracy could reflect the feasibility of detecting deception more clearly, and also provide a basis for detecting different deceptive individuals in practical applications. It examines the feasibility of the fNIRS technique to detect different deceptive individuals.

## Materials and Methods

### Participants and Protocol

Initially, 39 healthy adults participated in this study. Seven participants were excluded from further analysis because their data were missing or because they did not fully understand the experimental instructions. Finally, 32 valid participants were included (15 males and 17 females, aged 18–26, mean age 23.47 ± 2.21 years). All the participants had normal or corrected-to-normal vision, and no neurological or psychiatric diseases. Before the experiments, written, informed consent was obtained from all the participants. Our study was approved by the Ethics Committee of the School of Psychology at South China Normal University, and the methods were carried out in accordance with approved guidelines.

Our study used the paradigm of spontaneous deception, where the participants decide when and how many times to lie rather than guiding their behaviors (Chang et al., 2014; Panasiti et al., 2014), to increase the ecological validity. Self-related questions were adopted as the experimental materials, since individuals often lie more about themselves than others (Ganis et al., 2009). Moreover, self-related information is highly practiced and readily accessible (Nunez et al., 2005). Investigating self-related deception is critical for the practical application of lie detection.

Before the experiments, all the participants were asked to fill out a questionnaire which contained 64 self-related questions. They were required to answer these questions truthfully. Then, the participants were randomly assigned into the deceptive group (18 valid participants, regarded as “liars”) or the non-deceptive group (ND group, 14 valid participants, regarded as “innocents”). In each group, participants were instructed to answer self-related questions under two conditions: the task condition and the baseline condition. The orders of the task and the baseline condition were counterbalanced. Before the experiment, participants were told that they would get 20 RMB for payment. In the baseline condition, participants of both groups were required to answer the self-related questions truthfully all the time. In the task condition, a simulated task of detecting deception was conducted. Before this task, the two groups were given different instructions. Participants of the deceptive group were told to imagine the following situation: they were escaped prisoners, now under interrogation because they were suspects. A computer would record their answers, and they must hide their identity by deceiving the computer. They should answer those self-related questions with some strategy in the simulated task of detecting deception. That is, they needed to mix lies and truths, rather than tell lies all the time. They could decide when to lie and when to tell the truth spontaneously. Participants were also told that the computer would judge their identities at the end of the experiment. If the computer considered them escaped prisoners, they would lose 20 RMB for punishment. Correspondingly, participants of the ND group were required to imagine a situation: They were innocents, and now they were being interrogated because they were suspected as the escaped prisoners, so they should show their identities truthfully to convince the computer that they were not the escaped prisoners. They needed to answer those self-related questions truthfully all the time during the simulated task. They were told that if the computer considered them the escaped prisoners, they would lose 20 RMB. After the experiments, the judgment given by the computer would appear. In fact, every participant would be informed that they were determined to be innocent. After the experiments, the participants of the deceptive group were further divided into infrequently deceptive group (IFD group, regarded as “infrequent liars”) and frequently deceptive group (FD group, regarded as “frequent liars”) based on their number of lies in the task condition. Specifically, the top 50% of participants were defined as the FD group (9 valid participants) and the other half were defined as the IFD group (9 valid participants).

**Figure 1:**
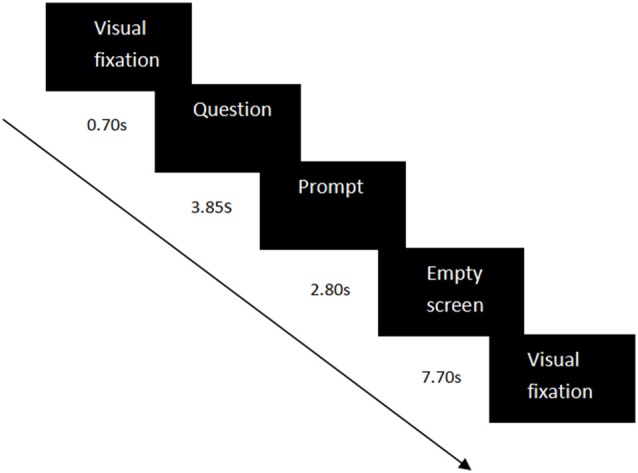
Experiment design in each experimental trial. Each trial contained a visual fixation (0.70 s), a question (3.85 s), a prompt (2.80 s), and an empty screen (7.70 s).

Sixty-four self-related questions were used as the experimental materials, including questions on semantic information (e.g., “Are you a student at South China Normal University?”) and questions on specific episodes (e.g., “Did you call your parents yesterday?”). Each condition contained 32 self-related questions—the number of questions on semantic information and on specific episodes were equal. The questions were the same for the three groups. The questions were set in a random order in each condition. In each trial, the visual fixation “+” appeared for 0.70 s to remind the participants to notice the center of the screen, then a self-related question was represented for 3.85 s. Next, a prompt was shown for 2.80 s to guide the participants to press the button. If their answers were “yes”, they should press “Q”; If their answers were “no”, they should press “P”. Eventually, an empty screen would appear for 7.70 s. The whole trial would last 15.05 s (see Figure 1). The time of each stage was set to a multiple of the temporal resolution (0.07 s) of the measurement of fNIRS.

### Experimental Setup

**Figure 2:**
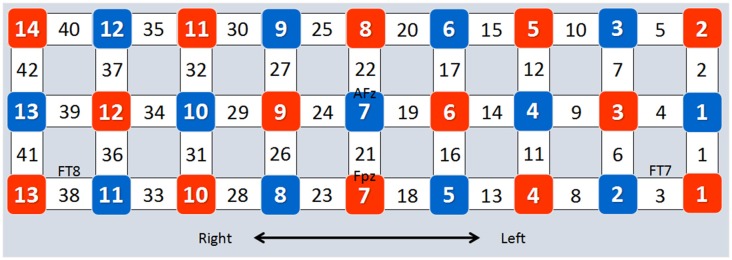
Optode placement and channel locations. The placement of the optodes is based on the four EEG sites (FPz, AFz, FT7 and FT8) of the 10–10 system. Red squares represent emitters, blue squares represent detectors, and numbers in blank squares represent channel numbers.

Forty-two channels of an fNIRS system (FOIRE-3000, Shimadzu Corporation, Kyoto, Japan) were used in the present study (Kajimura et al., 2014). This system operates at three wavelengths (780 nm, 805 nm and 830 nm; Zhu et al., 2014). Concentration changes of oxygenated hemoglobin (HbO) and deoxygenated hemoglobin (Hb) were measured simultaneously, and changes of total hemoglobin (HbT) were calculated by adding HbO and Hb (Chang et al., 2014). Optical data were transformed into HbO and Hb according to the modified Beer-Lambert Law (Baker et al., 2014). The optode replacement and the locations of the channels are presented in Figure 2. According to the 10–10 system (Koessler et al., 2009), channels 8, 16, 25 and 33 were associated with the right IFG, while channels 1, 10, 18, 27 were associated with the left IFG, and channels 11, 19, 20, 28, 36 and 37 were associated with the left MFG.

### Data Analysis

The data from effective experimental trials were selected as described below. Trials where the behavioral data were not recorded were excluded from further analysis. In the baseline condition of the three groups, the trials where the answers were not consistent with the questionnaire were excluded. In the task condition of the IFD group and the FD group, the trials where the answers were consistent with the questionnaire were excluded. In the task condition of the ND group, the trials where the answers were not consistent with the questionnaire were excluded. The remaining trials contained the truth-telling trials in the baseline condition of the three groups, the lying trials in the task condition of the IFD group and the FD group, and the truth-telling trials in the task condition of the ND group. Thus the baseline data were from truthful statements of the baseline conditions in the three groups, contrasted against task data from truthful statements of the task condition in the ND group and lies in two deceptive groups.

When processing the fNIRS data, group analysis and individual analysis were both used. These analysis were completed by NIRS-SPM (Ye et al., 2009) and SPSS 19.0. The HbO data and the Hb data were both analyzed. However, if the results of HbO analysis and Hb analysis were different, we prioritized HbO results because HbO signals are the most sensitive index to reflect cerebral blood flow activities, whereas the Hb signals are relatively noisy and unreliable (Ding et al., 2014).

Before group analysis, general linear model (GLM) analysis was performed for each participant. In GLM, observed data, such as hemodynamic response in a channel (dependent variable), are defined as a linear combination of predictor variables (independent variables) plus an error term (The formulation is *y_i_* = *β*_1_*X*_i1_ + *β*_2_*X*_i2_ + … + *β*_j_*X*_ij_ + *ε*_i_, where *y*_i_ represents observation i, *X*_ij_ represents value i for predictor variable j, *β*_i_ represents parameter estimated for predictor variable j, *ε*_i_ represents error for observation i.) (Suryakumar et al., 2007; Jang et al., 2009). GLM is generally used in fMRI studies (Friston et al., 1994). In this study, GLM can describe a measurement of change in HbO/Hb in terms of a linear combination of two predictor variables (the task condition and the baseline condition), so the beta values can be explained as the relationship between change in HbO/Hb and specific experimental tasks. In fact, beta values of the GLM for different conditions can be extracted as weights to account for the brain activity. The GLM analysis was performed as the following two steps: first, for each participant, the hemodynamic response function (HRF) filter and a wavelet-MDL (minimum description length) detrending algorithm were used to remove physical noise and artifacts, and a baseline correction was executed. Wavelet-MDL (minimum description length) detrending algorithm was utilized to decompose fNIRS measurements into global trends (including subject movement, blood pressure variation and/or instrumental instability), hemodynamic signals and uncorrelated noise components on distinct scales (Jang et al., 2009). After the wavelet-MDL based detrending, the average HbO time series were estimated by integrating each HRF with the relevant experimental paradigms. This method could improve the signal-to-noise ratio, and output more specific activation signals than a traditional method such as simple filtering (Jang et al., 2009; Zhu et al., 2015). Second, all the data points within 15.05 s from each effective trial in the task condition and the baseline condition were used to estimate the beta values of GLM for each participant. The mean baseline length of each participant was 458.08 s (mean 30.44 trials).

Group analysis was then conducted after GLM analysis: (1) based on the beta values, HbO and Hb maps of mean values were depicted (Matlab codes are shown in Supplementary Data Sheet 1). (2) ROIs of the brain were selected based on the HbO and Hb maps. Because inhibition function during deception was our central focus, only the obviously activated channels of the left MFG or the bilateral IFG were the candidates for ROIs. For HbO maps, obvious activation was a beta value >0.018, while for Hb maps, obvious activation was a beta value <−0.01. (3) A three-way repeated measures analysis of variance (ANOVA) test (ROI * group * condition) was conducted to examine the differences in beta values in two conditions among three groups (if there was only one ROI, a two-way ANOVA of group * condition was performed). Time course waveform analysis was performed according to the following steps: (1) the data after baseline correction were transformed to a *Z*-score representation. (2) For each participant, all effective trials of the task condition and all effective trials of the baseline condition were separately averaged in each channel. (3) For each group, the data of the task condition and the baseline condition were separately averaged across corresponding participants. Thus, the mean time course waveform of each channel for each condition in each group was derived (Matlab codes are shown in Supplementary Data Sheet 1). Only the data of ROIs that showed significant results in the ANOVA analysis are considered in the time course waveform analysis.

The individual analysis was performed as follows: first, detection regions were selected based on the group analysis. Secondly, receiver operating characteristic (ROC) analysis and support vector machine (SVM) analysis were both conducted to detect the accuracy in differentiating infrequent liars from innocents, and in differentiating frequent liars from innocents (Sai et al., 2014). The specific methods shall be discussed later.

## Results

### Group Analysis

#### HbO Data

**Figure 3:**
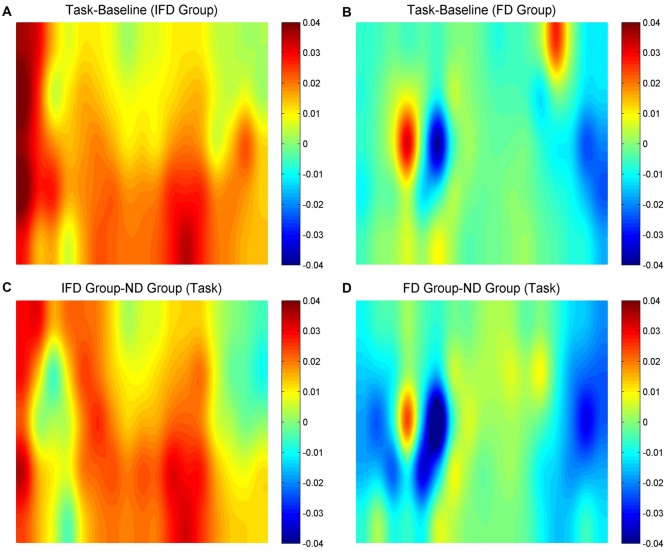
The oxygenated hemoglobin (HbO) maps.** (A,B)** represent “the task conditions minus the baseline condition” of the IFD group and the FD group, **(C)** represents “the IFD group minus the ND group” in the task condition, and **(D)** represents “the FD group minus the ND group” in the task condition. The channel locations are the same as in Figure 2.

HbO maps of “the task condition minus the baseline condition” in two deceptive groups, the task condition of “the IFD group minus the ND group” and the task condition of “the FD group minus the ND group” are all shown in Figure 3. Because the focus of our study was the inhibition-related brain regions, we only paid attention to the activation of the channels from the left MFG and the bilateral IFG. The candidates of ROIs should meet two conditions: the activation of “the task condition minus the baseline condition” in either deceptive group should be obvious and the task condition of “either deceptive group minus the ND group” should be obvious. As the HbO maps show, among all the channels in the bilateral IFG and the left MFG, channel 11 and channel 20 (both in the left MFG) both met the two conditions. Thus, channel 11 and channel 20 were selected as ROIs in the HbO analysis.

**Figure 4:**
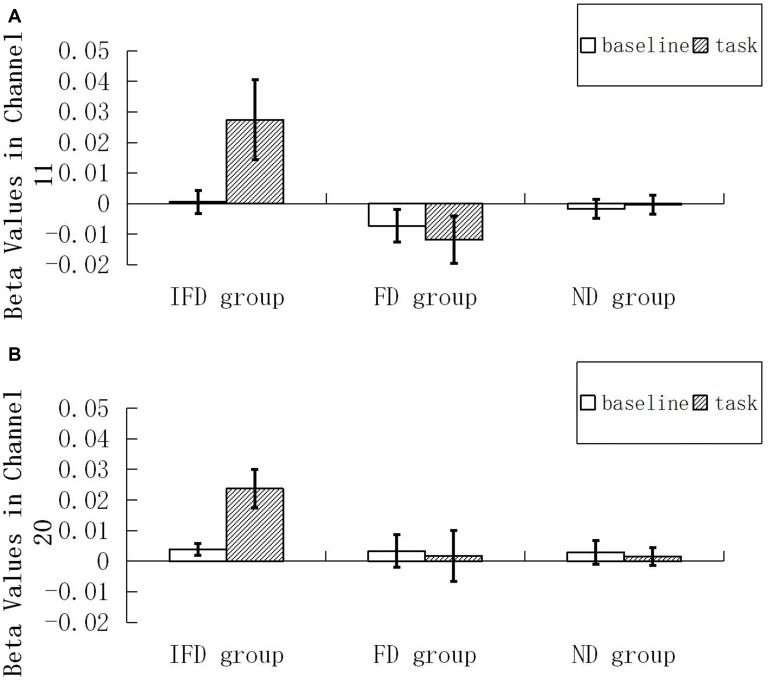
Beta values of HbO. Mean beta value of the baseline condition and the task condition among the IFD group, the FD group and the ND group. **(A,B)** represent beta values in channel 11 and channel 20.

For the HbO data, we performed 2 (ROI) * 2 (condition) * 3 (group) repeated measures ANOVA. The main effect of condition was significant (*F*_(1,29)_ = 5.035, *p* = 0.033, $\eta_{\text{p}}^{\text{2}}$ = 0.148), the main effect of group was significant (*F*_(2,29)_ = 4.441, *p* = 0.021, $\eta_{\text{p}}^{\text{2}}$ = 0.234), and the interaction effect of condition and group was significant (*F*_(2,29)_ = 7.153, *p* = 0.003, $\eta_{\text{p}}^{\text{2}}$ = 0.330). Simple effect analysis indicated that, when performing the task condition, the IFD group exhibited a significantly greater increase in HbO than both the FD group (*p* = 0.005) and the ND group (*p* = 0.012). In addition, in the IFD group, the task condition led to a significantly greater increase in HbO than the baseline condition (*p* = 0.0002). However, in the FD group and the ND group, differences in changes in HbO of the task condition and the baseline condition were not significant (*p*_min_ = 0.578). Additionally, the main effect of ROI and other interaction effects were all not significant (*p*_min_ = 0.085; see Figure 4).

**Figure 5:**
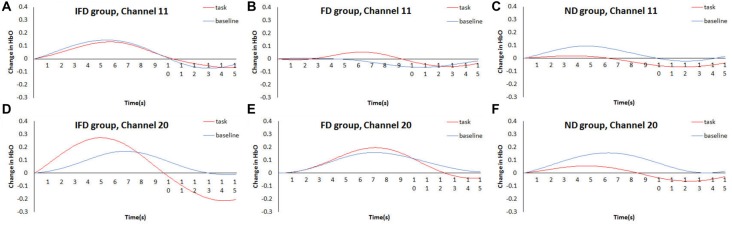
Time courses waveform of HbO changes. The time courses of the mean HbO changes (*Z* value) of the task condition and the control condition of three groups. **(A–C)** represent HbO changes of IFD group, FD group and ND group in channel 11, **(D–F)** represent HbO changes of IFD group, FD group and ND group in channel 20.

The data of channel 11 and channel 20 were analyzed by time course waveform analysis (Figure 5). Considering different hemodynamic responses owing to task preparations of the task condition and the baseline condition (Jamadar et al., 2010; Ito et al., 2012), the HbO waveforms of these two conditions were both set to start from zero on the *y* axis (see Supplementary Data Sheet 2,3). In channel 20, under the task condition of the IFD group, obvious HbO growth was observed approximately from 4.5 s to 7 s (the period of executing deceptive behavior). Also, during the same period, this HbO signal was greater than the baseline condition of the IFD group, as well as greater than the task conditions of FD group and ND group. However, this pattern was not observed in channel 11.

Additionally, a 2 (ROI) * 3 (group) ANOVA was conducted to examine the error differences estimated by GLM among three groups. Results showed that the main effect of group and the interaction effect of ROI and group were not significant (*p* = 0.183, 0.924), which indicated that there was no significant error differences among three groups. We also checked mean *Z*-scores of the HbO data in channel 11 and channel 20 from each trial. We found that no data point was out of three standard deviations above the mean (|*Z*|_max_ = 2.83), indicating that there were no extreme values in IFD group and FD group. In summary, group differences are unlikely to be an artifact of systematic differences in noise.

#### Hb Data

**Figure 6:**
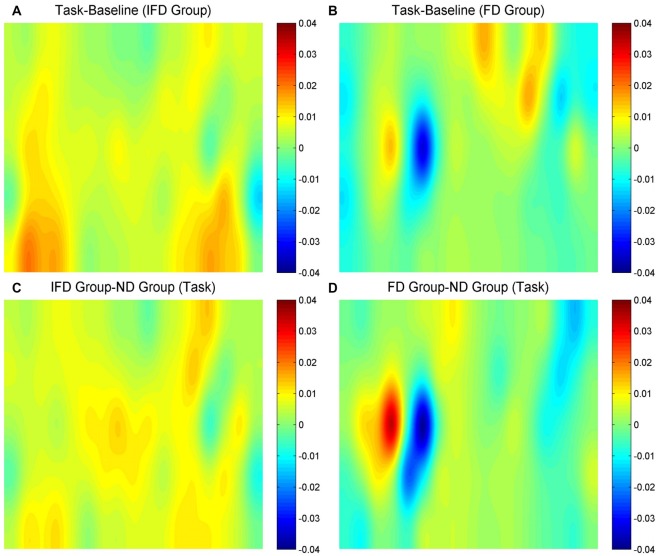
The Hb maps.** (A,B)** represent “the task conditions minus the baseline condition” of the IFD group and of the FD group, **(C)** represents “the IFD group minus the ND group” in the task condition, and **(D)** represents “the FD group minus the ND group” in the task condition. The channel locations are the same as in Figure 2.

Hb maps of “the task condition minus the baseline condition” in two deceptive groups, the task condition of “the IFD group minus the ND group” and the task condition of “the FD group minus the ND group” are all shown in Figure 6. The standards of selecting ROIs were the same as for HbO analysis. As the Hb maps show, among all the channels in the bilateral IFG and the left MFG, the activation of channel 27 was obvious in “the task condition minus the baseline condition” of the FD group, as well as in the task condition of “the FD group minus the ND group”. Thus, we selected channel 27 as the only ROI in the Hb analysis.

For the Hb data, we performed a repeated measures 2 (condition) * 3 (group) ANOVA in channel 27. Results showed that the main effect of the condition, the main effect of the group, and the interaction effect of condition and group were all not significant (*p*_min_ = 0.409). Because this channel did not show significant results, we do not present the time course waveform analysis. Also, we did not include Hb data in individual analysis.

### Individual Analysis

According to the group analysis, neural activities of deception were significantly greater than the baseline from HbO data in the MFG. In two ROIs, only the data of channel 20 met the standards of differentiating liars from innocents in time course waveform analysis. Thus we selected channel 20 as the detection region.

#### ROC Analysis

**Figure 7:**
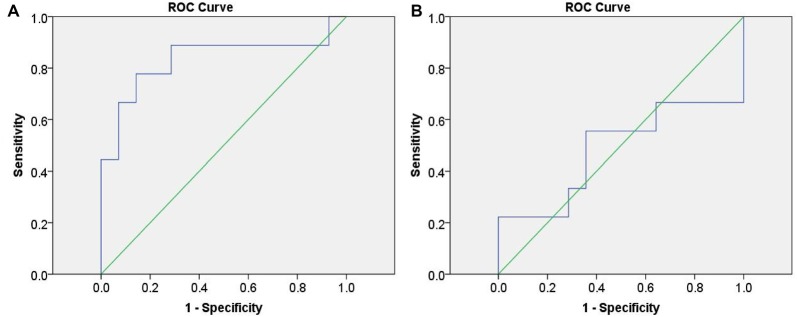
Receiver operating characteristic (ROC) curves based on functional near-infrared spectroscopy (fNIRS) data.** (A)** represents differentiating infrequent liars from innocents and **(B)** represents differentiating frequent liars from innocents.

Initially, we calculated the values of change in HbO from “the task condition minus the baseline condition” of the three groups in channel 20. These data were set as the index to discriminate between infrequent liars and innocents, as well as between frequent liars and innocents. The ROC curves are shown in Figure 7.

ROC analysis indicated fNIRS data could differentiate infrequent liars from innocents at 83.3% accuracy (*AUC* = 0.833 (0.633–1.000), *p* = 0.008). However, it could not differentiate between frequent liars and innocents above a chance level (*AUC* = 0.484 (0.209–0.759), *p* = 0.900).

#### Support Vector Machine (SVM) Analysis

Support Vector Machine (SVM) analysis was performed by the following steps: the beta values of the task condition and the baseline condition in channel 20 were both included in a SVM algorithm to build a classifier between liars and innocents. Nine participants of IFD group and 14 participants of ND group were set as Sample 1 to differentiate between infrequent liars and innocents, and nine participants of FD group and 14 participants of ND group were set as Sample 2 to differentiate between frequent liars and innocents. Sixteen participants were randomly selected for training and the left seven participants for predicting in both samples. This program was repeated 1000 times for cross validation.

Results showed that, when channel 20 was set as the detection index, the accuracy of differentiating infrequent liars from innocents was 78%. Specially, the sensitivity of deception detection was 66.67% and the specificity was 88.37%. The accuracy of differentiating frequent liars from innocents was 69.86%. The sensitivity of deception detection was 50% and the specificity was 85.3%.

## Discussion

The feasibility of fNIRS monitoring of inhibition-related brain regions to detect both infrequent liars and frequent liars was considered. We compared the strength of the neural activities of these two types of deceptive individuals at the group level, then analyzed the accuracy of detecting deception at the individual level.

Our study found that frequency of deception could affect the inhibition-related brain responses to deception from group analysis. Specifically, we found that during deceiving, frequent liars showed less hemodynamic activation than infrequent liars in the left MFG. This result was consistent with Jiang et al.’s (2013) study. For the infrequent liars, deception is not a dominant response, so they require greater cognitive effort to inhibit the habitual truthful response. We observed that, from the results of time course waveform analysis, infrequent liars showed an HbO response reaching a peak at about 5 s while deceiving, within the stage of producing deceptive answers. This phenomenon suggests that the process of inhibition occurs during the stage of deceiving execution rather than the preparation stage. In addition, previous studies suggests that frequent deception makes deceiving easier (Verschuere et al., 2011; Van Bockstaele et al., 2012). Since it is their habitual response, frequent liars do not require as much cognitive effort to inhibit the truth as infrequent liars. In fact, inhibition-related brain regions are the domain-general areas whose functions would be modulated by individual variability to a large extent. Several past studies have confirmed this phenomenon. For instance, Marchewka et al. (2012) revealed that gender had an influence on the neural signals of the inhibition-related areas. Women would exhibit less activation in the left MFG than men when they deceived.

The results of group analysis indicated that, compared to the baseline condition, infrequent liars showed significantly greater neural activities in the left MFG during deceiving. One interesting result was that this difference did not apply to the activation in the bilateral IFG. A possible interpretation is that, IFG appears to be a special area involved in inhibition (Hampshire et al., 2010), so it might be activated when the process of inhibition were the primary cognitive activity. However, our paradigm of spontaneous deception involved multiple mental activities such as risk taking, mentalizing and inhibiting the truth (Sip et al., 2008; Spence et al., 2008; Christ et al., 2009; Leue et al., 2012; Ding et al., 2014), thus deceiving tended to activate the left MFG rather than IFG. The other interesting result was that, from the results of time course waveform analysis, the difference between lying behaviors and baseline among infrequent lairs reflected in only one channel in two ROIs. Because the effect of task preparation was not considered by time course waveform analysis, we speculate that this difference will decrease when examining simple neural activity associated with answering questions in deception detection tasks. Furthermore, frequent liars did not show any significant activation of the inhibition-related regions (involving the left MFG and the bilateral IFG) compared to the baseline. This finding implies that frequent liars not only need little energy to inhibit the truth, but also execute the deceptive response as if they are telling the truth (Blair et al., 1997). Additionally, different from many previous studies, we examined the neural activities of innocents, rather than just the liars (Jiang et al., 2013; Li et al., 2015). We found that innocents did not manifest significantly greater neural activation than baseline in any part of inhibition-related regions while performing deception detection tasks. These results illustrated that infrequent liars and innocents show distinct neural activation patterns in inhibition-related regions during the deception detection tasks, indicating that infrequent liars could be separated from innocents. However, due to similar activation patterns, frequent liars are indistinguishable from innocents.

Individual analysis indicated that frequency of deception could have an effect on the accuracy of detecting deception. For ROC analysis, our results showed that fNIRS could differentiate infrequent liars from the innocents at an accuracy with 83.3%, while it could not successfully distinguish the frequent liars from the innocents above a chance level. Moreover, SVM analysis indicated that, using the left MFG as the detection region, 78% classification accuracy, as well as 66.67% sensitivity of deception detection, could be achieved when detecting infrequent liars. However, when detecting frequent liars, classification accuracy was lower than 70%, and the sensitivity of deception detection declined significantly. Combined with ROC analysis and SVM analysis, our study indicated that above-chance accuracy could be obtained when differentiating the infrequent liars from innocents. Moreover, it suggests that the detection index has a moderate ability to distinguish between deceiving and telling the truth in infrequent liars. In contrast, when differentiating the frequent liars from innocents, acceptable accuracy could not be achieved. We could not find out the differences between lying responses and truthful responses from any frequent liar.

In practical applications, the index of inhibition-related regions should be used with great caution in detecting various liars. We propose that two possible measures could improve the ability to detect frequent liars. First behavioral analysis could be adopted as a supplementary method when using the fNIRS technique. Despite the mainstream view that the neural signal of deceiving should be more reliable (Bhutta et al., 2015), behavioral analysis combined with neural activity analysis might provide a more comprehensive view of the deception process. In fact, previous fNIRS study has verified that combined indices (fNIRS data and behavioral data) could improve the accuracy of lie detection beyond simple fNIRS index (Sai et al., 2014). Secondly, not only the regions involved in inhibition, but also regions associated with other cognitive activities during deception should be examined by fNIRS. For instance, even though frequent liars do not need much effort to inhibit the truth, they still need effort to consider their strategy of deceiving. This process is strongly linked to the function of planning (Ding et al., 2014). Planning is thought to be typically associated with the function of the superior frontal gyrus (SFG; Baker et al., 1996), so it is plausible that bringing SFG into the detection index might enhance the ability of fNIRS to detect frequent liars. Since more social cognition are engaged in interpersonal interaction (Volz et al., 2015), interrogation could be conducted more frequently by a human than by a computer in the future.

## Conclusion

In summary, our study has indicated that using inhibition-related brain regions to detect deception is feasible for infrequent liars and not feasible for frequent liars.

## Author Contributions

FL and SH designed the experiment. FL analyzed and interpreted the data, and wrote the manuscript. HZ provided technical support for analyzing and interpreting the data, and helped write and revise the manuscript. SH, HZ and JX revised the manuscript. FL, JX, QG, HG, SW and XL performed laboratory work and collected the data. All the authors read and approved the final manuscript.

## Conflict of Interest Statement

The authors declare that the research was conducted in the absence of any commercial or financial relationships that could be construed as a potential conflict of interest.
